# Measles Population Immunity Profiles: Updated Methods and Tools

**DOI:** 10.3390/vaccines12080937

**Published:** 2024-08-22

**Authors:** Xi Li, James L. Goodson, Robert T. Perry

**Affiliations:** Accelerated Disease Control Branch, Global Immunization Division, Centers for Disease Control and Prevention, Atlanta, GA 30329, USA

**Keywords:** measles, vaccination, immunity, risk assessment, coverage, mathematical model

## Abstract

Measles is a highly contagious disease and remains a major cause of child mortality worldwide. While measles vaccine is highly effective, high levels of population immunity are needed to prevent outbreaks. Simple but accurate tools are needed to estimate the profile of population measles immunity by age to identify and fill immunity gaps caused by low levels of vaccination coverage. The measles immunity profile estimates and visualizes the percentage of each birth cohort immune or susceptible to measles based on measles vaccination coverage. Several tools that employed this approach have been developed in the past, including informal unpublished versions. However, these tools used varying assumptions and produced inconsistent results. We updated the measles population immunity profile methodology to standardize and better document the assumptions and methods; provide timely estimates of measles population immunity; and facilitate prompt actions to close immunity gaps and prevent outbreaks. We recommend assuming that the second dose of the measles-containing vaccine (MCV2) and doses given during supplementary immunization activities (SIAs) first reach children who have been previously vaccinated against measles, so that previously unvaccinated children are reached only when the coverage of MCV2 or SIA is higher than the coverage achieved by all previous measles vaccination opportunities. This updated method provides a conservative estimate of immunization program impact to assess measles outbreak risk and to facilitate early planning of timely preventive SIAs to close population immunity gaps.

## 1. Introduction

Measles remains a major cause of child mortality, despite being preventable by vaccination: in 2022, an estimated 136,200 measles deaths occurred worldwide, mostly among children less than 5 years of age [1]. The measles virus is one of the most contagious human pathogens known with a basic reproductive number (R_0_) of 9–18 [2,3]. As a consequence of high transmissibility of the measles virus, high population immunity (estimated to be 89–94%) is needed in order to achieve herd immunity and prevent large and disruptive outbreaks of measles [4]. The exact threshold for herd immunity varies depending on context-specific determinants, such as birth rate, population density, age-specific contact rates, and population mixing. Measles can be prevented by using measles-containing vaccines (MCVs), which are safe and effective. The World Health Organization (WHO) recommends that all countries provide two doses of a measles-containing vaccine (MCV) in their national immunization schedule during infancy and early childhood [5]. Implementing periodic mass vaccination campaigns, also referred to as supplementary immunization activities (SIAs), is an effective strategy to close population immunity gaps by delivering the second dose (if not already introduced into the routine schedule), catching up children missing the first or second dose, and reaching hard-to-reach children. WHO recommends that countries conduct SIAs until >90–95% coverage has been achieved for both the first and second dose of a measles-containing vaccine (MCV1 and MCV2, respectively) in the national routine immunization schedule [5]. Nationwide preventive SIAs aim to vaccinate all children in the country in the target age group, regardless of their previous vaccination history. SIAs should be conducted regularly when the number of measles-susceptible children less than 5 years of age approaches the size of one birth cohort [5].

Global progress has been made toward reducing measles incidence and mortality and achieving measles elimination. During 2000–2022, global measles mortality was reduced by 82% and measles incidence decreased by 75% [1]. In 2010, at the World Health Assembly (WHA), countries endorsed the target to increase MCV1 to ≥90% at national level and ≥80% in every district, reduce global annual measles incidence to less than 5 cases per million population, and reduce global measles mortality by 95% from the 2000 estimate by 2015 [6]. In 2012, the WHA endorsed a resolution to eliminate measles in five of the six WHO regions by 2020 [7]. As of the end of 2022, 83 (43%) countries have achieved measles elimination [1]. However, in recent years, there have been large measles upsurges. In 2019, the global measles incidence was 120 cases per million population, representing a 567% increase compared to 18 cases per million in 2016, the year with the lowest incidence during 2000–2019 [1]. By 2021, global measles incidence decreased by 86% from 2019 to 17 cases per million population, but increased 71% to 29 cases per million population in 2022, although only 43% of countries reported an adequate incidence of febrile rash illness for which measles has been ruled out by laboratory testing, indicating inadequate surveillance sensitivity globally [1]. Global MCV1 coverage decreased from 86% in 2019 to 81% in 2021, and then increased to 83% in 2022 [1]. As a result of immunization service interruptions due to the COVID-19 pandemic, 41 SIAs were delayed; of these, 35 had been completed by the end of 2022 [1].

Estimating measles population immunity gaps can help guide immunization program managers using data to monitor program performance, identify the timing for the next SIAs, and identify age groups with large measles immunity gaps that need to be included in the SIA target age group. Serosurveys provide direct estimates of measles immunity through the collection of biological specimens such as blood and saliva from participants and testing for measles-specific immunoglobulin G (IgG) levels; the percentage of a population susceptible to measles is defined by the percentages of persons negative for measles IgG by age group. If serosurveys are geographically representative and well implemented, then they provide a valid empirical measurement of the population immunity gaps. However, serosurveys are costly and conducted infrequently, only provide a cross-sectional snapshot of measles immunity at the time when conducted, and often do not provide timely results for programmatic decision-making given the time for specimen collection, transport to the lab, testing, and statistical analysis. Estimating population immunity using readily available data, namely, MCV1, MCV2, and SIA coverage, can provide more timely estimates on measles population immunity gaps. Measles immunity profiles show the percentage of persons immune as a result of routine immunization with MCV1, routine immunization with MCV2, and vaccination through SIAs, and the percentage of persons susceptible to measles by each year’s birth cohort based on the analysis of vaccination coverage data that are publicly available, including the WHO and United Nations Children’s Fund (UNICEF) Estimates of National Immunization Coverage (WUENIC).

Several tools have been previously developed to generate measles immunity profiles. Since the 1990s, multiple informal tools have been developed using different versions of Microsoft Excel and used by countries and partners. These spreadsheets included varying assumptions and formulas, did not clearly document the methods and assumptions, and often generated inconsistent results. Analytical results of these previously existing informal tools, named susceptibility profiles, have been used for programmatic decision-making and occasionally were included in published articles [8,9]. To provide a more standardized and documented approach, WHO joined with PATH and Stanford University to develop the Measles Strategic Planning (MSP) tool. This tool was designed to show the impact on immunity, cases, and deaths of various options to accelerate measles control effort [10]. The MSP tool provides measles immunity profiles for the baseline and final year of the modeled period and shows the percentage of persons immune as a result of routine immunization with MCV1 and MCV2, and vaccination through SIAs and measles infection, and the percentage of persons who remain susceptible for each birth cohort. One key assumption of the MSP tool was independence between MCV1 and SIA doses, which meant that every child in a country had the equal chance to be vaccinated with each SIA dose regardless of whether they had been previously reached by immunization services. This approach predicted small immunity gaps in age groups that contributed significant numbers of cases according to surveillance data. As detailed below, we recommend using the assumption that children who have been previously vaccinated have higher chances of being reached by routine immunization with MCV2 and by SIAs in order to provide a conservative estimate of immunization program impact, which aids early and timely planning of SIAs. We have developed a set of tools based on Microsoft Excel and a program written in the R programming language with the following objectives: (1) standardize and better document assumptions and methods; (2) provide timely estimates of population measles immunity levels to facilitate prompt actions to close immunity gaps and prevent outbreaks.

## 2. Method

### 2.1. Tool Development Process

We reviewed the methods, assumptions, and input parameters for several informal versions of measles immunity profile tools and the MSP tool. Subject matter experts were consulted to gain consensus on choices of methods and parameters that were then presented to the WHO Immunization and Vaccines Related Implementation Research Advisory Committee for review in August 2021 [11].

### 2.2. Input Data

The input data of the immunity profiles include coverage of MCV1, MCV2, and SIAs, the target population is relevant for SIAs, and population size by age. For MCV1 and MCV2, we used WUENIC as they are based on administrative data, surveys, and information on routine immunization program performance (strikes, stockouts, etc.) and updated annually for all WHO member states. Estimates of coverage reached by SIAs were based on post-campaign coverage surveys (PCCSs) whenever available, or if a survey coverage estimate was not available, then they were based on estimates from administrative data. Administrative coverage for SIAs was capped at 95% if not supported by a PCCS, as very high administrative coverage is usually an indication of poor data quality and to avoid coverages greater than 100%.

We used population estimates of the number of persons by each one-year of age (i.e., <1 year, 1 year to less than 2 years, etc.) from the World Population Prospects, 2019 Revision published by United Nations Population Division.

### 2.3. Key Assumptions and Formulas

#### 2.3.1. Association between Doses

WHO recommends that an administered dose of MCV should be recorded as MCV2 only if the child has the documented first dose of MCV1 [12,13]. In an ideal routine immunization services delivery program, all children coming for measles vaccination will be screened and receive MCV1 or MCV2 based on information in the clinic’s vaccination register or the home-based record. Therefore, the formulas use the assumption that the children receiving the second dose would only be those who have already received the first dose; that is, MCV2 is dependent on MCV1. However, in the real world, some children coming for the MCV2 visit may not have received MCV1 but may be accidently recorded as having received MCV2 during the visit, not MCV1, because the child’s age is at or past the age when they were expected to receive MCV2. MCV1 coverage may be lower than MCV2 in some situations, for example, when doses given at an older age are recorded as MCV2 without checking the child’s MCV1 status. Furthermore, MCV1 and MCV2 reported in the same year are often given to different birth cohorts, so for some years in some countries, the MCV2 estimate is higher than that for MCV1.

During each SIA, if the chance of being vaccinated is the same both for children who have previously received an MCV and for children who have not received an MCV, then the chance of vaccination is independent of the vaccination status before the SIA (the “independent scenario”). This assumption contrasts with the common observation that vaccinated children have better access to health services and these children are generally more reachable by subsequent SIAs. In addition, SIAs will also reach some previously unvaccinated children because of SIA strategies such as increased outreach, social mobilization, communications, and resources designed to reach previously unvaccinated children. To better capture the dependency between vaccination opportunities, we assumed that each vaccination opportunity reaches all children who have been vaccinated before, and then, only if the vaccination coverage is higher than previous vaccination opportunities, will the SIA reach and vaccinate previously unvaccinated children (the “dependent scenario”). The “independent scenario” and “dependent scenario” represent the boundaries of a range where the real-world association between previous vaccination status and the probability of receiving a subsequent dose lies. As noted below, some previously vaccinated children may still not be immune, and we assume that revaccination will provide these children with a second opportunity for immunity.

**Figure 1:**
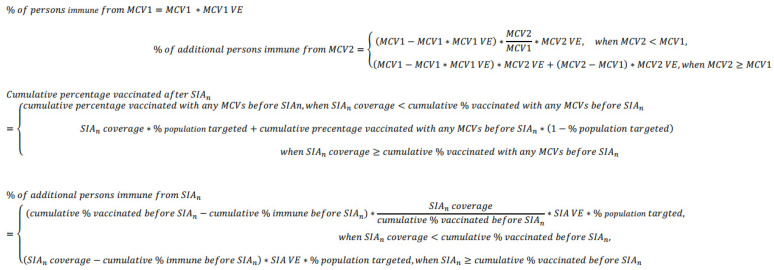
Measles population immunity profile method formulas using the dependent assumption that previously vaccinated children are first reached by a subsequent vaccine dose. Notes—MCV: measles-containing vaccine. MCV1: coverage of the first dose of MCV delivered through routine immunization; MCV2: coverage of the second dose of MCV delivered through routine immunization; SIA: supplementary immunization activity; VE: vaccine effectiveness.

We recommend using the “dependent scenario” to provide a conservative estimate of immunization program impact, and to reduce the chance of overestimating population immunity and delaying vaccination activities. Formulas using the dependent assumption are shown in Figure 1.

#### 2.3.2. Impact of Subnational SIAs

To account for subnational SIAs, we estimated the additional percentage of the national population immune to measles by multiplying the additional percentage of children who gained measles immunity through the SIA in the targeted subnational area by the estimated proportion of the country’s population living in that same subnational area.

#### 2.3.3. Vaccine Effectiveness

The vaccine effectiveness (VE) of a measles vaccine dose is dependent on the age of administration and is measured by comparing disease rates in vaccinated vs. unvaccinated people, as compared to the proportion of previously seronegative people with adequate immunity after vaccination [14]. A literature review suggested the median VE of a single dose of MCV1 to be 84% if administered at 9–11 months of age, and 92.5% if administered at 12 months and above [15]. Among children who do not develop immunity after MCV1, approximately 95% will develop immunity with a second dose [5]. A second literature review estimated the MCV1 VE for children vaccinated before 9 months of age to be 58% [16]. Our assumptions of the VE of MCV1 and MCV2 were based on the recommended age of vaccine administration in the national immunization schedule. For routine immunization, vaccines were assumed to be given at the earliest ages in the national immunization schedule. SIAs provide vaccinations for people of a defined age range, typically children, based on the rate of accumulation of measles-susceptible persons to prevent outbreaks, or to respond to outbreaks. Age-specific estimates of vaccine effectiveness were applied to SIA doses and age-disaggregated SIA vaccination coverage data were used whenever available.

#### 2.3.4. Cohort Granularity

In previous Excel-based measles population immunity profile tools, calculations were limited to whole calendar year birth cohorts, which means that the entire yearly cohort is either covered or not covered by an SIA. In the R-based measles immunity profile tool, cohorts were constructed based on the exact dates of SIAs and each cohort has unique experiences of MCV1 coverage, MCV2 coverage, SIA coverage, and vaccine effectiveness at the time of vaccine administrations. The percentage of persons immune to measles is calculated for each birth cohort and the results could be aggregated by age in years or by calendar year. This approach allows more accurate estimates of the youngest and oldest age groups in cohorts covered by an SIA, and more accurate calculations using age-specific vaccine effectiveness inputs.

#### 2.3.5. Date of the Profile

The profiles may be constructed at any date, as long as input data are available. When constructed for a future date, the profile could show the immunity gaps by cohort before a planned SIA, or the increase in population immunity after a planned SIA.

## 3. Results

We developed a set of tools to generate and visualize measles population immunity profiles to suit the needs of users with different software skill levels. The calculations were implemented through either a Microsoft Excel 365 spreadsheet or a program written in the R language. The calculations using R generate more accurate estimates than the calculations possible in Excel as more granular analyses could be performed in R, whereas in the Excel spreadsheet, one row represents each whole calendar year birth cohort. The key assumptions and characteristics for the calculations in Microsoft Excel and in R are shown in Table 1. A Power BI dashboard was developed to visualize the results of the R programs and an online, web-based Shiny R dashboard was developed for users to review and revise the input data and perform the calculations based on the formulas in R codes. The codes were annotated in a Jupyter notebook.

National-level measles immunity profiles have been developed for global, regional, and national partners and programs. A few examples of use cases are listed below.

### 3.1. Identify Birth Cohorts with Measles Immunity Gaps

**Figure 2:**
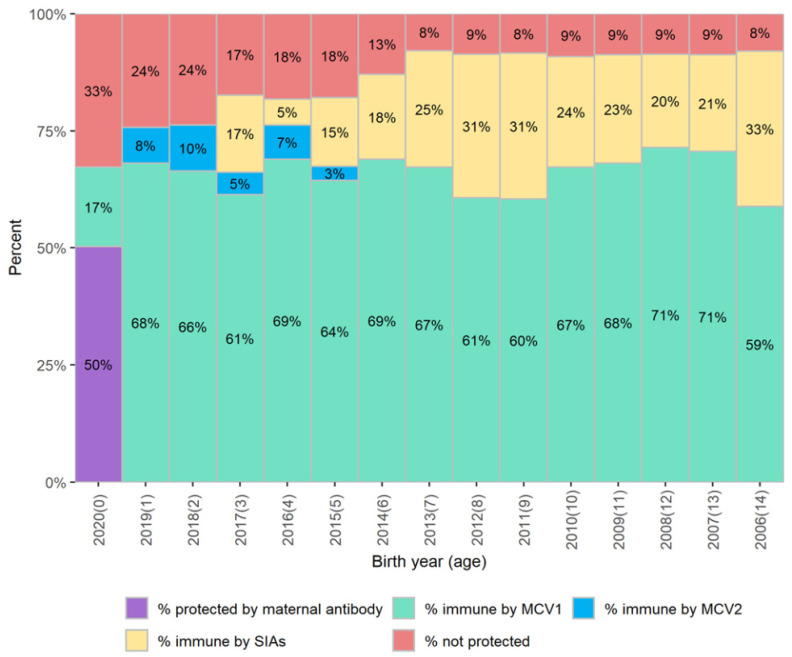
The measles population immunity profile as of 31 December 2020 showing estimated measles immunity gaps in red vertical bars for each birth cohort in a hypothetical country. Notes—MCV1: the first dose of measles-containing vaccines; MCV2: the second dose of measles-containing vaccines; SIA: supplementary immunization activity.

The measles population immunity profile graph visualizes immunity by birth cohort. Figure 2 shows an example of an immunity profile as of 31 December 2020 in a hypothetical country with an annual birth cohort of 1 million infants. The immunization coverage data for MCV1 and MCV2 delivered by routine immunization and data for MCVs delivered by SIA in this hypothetical country are shown in Table 2 and Table 3. The profile indicates that among children born since 2014, >10% of children in each birth cohort are not protected against measles by vaccination, as a result of suboptimal routine immunization coverage and the suboptimal coverage of the last SIA. Achieving higher coverage through routine immunization and a high-quality SIA among the cohorts with large population immunity gaps would be needed to close the immunity gaps.

### 3.2. Estimate Impact of a Planned SIA or Change in Routine Immunization Coverage

**Figure 3:**
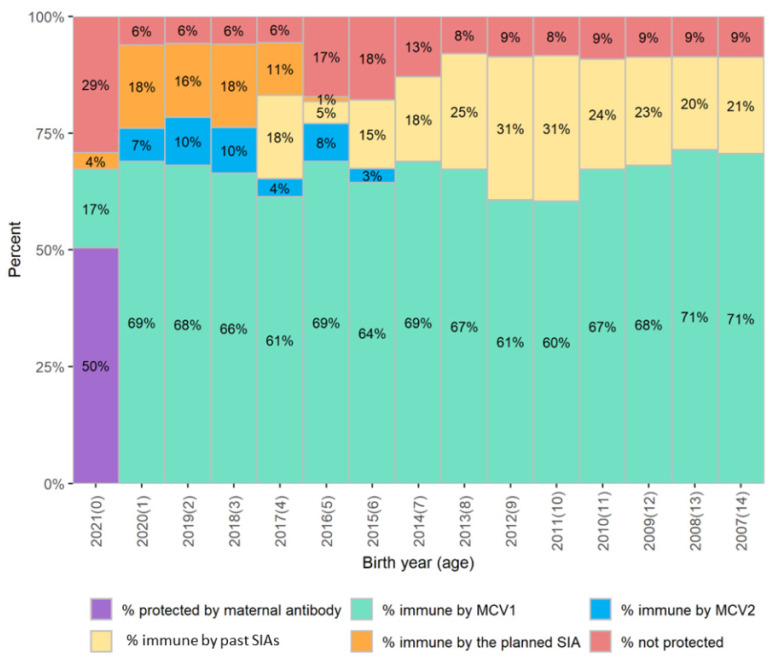
The measles population immunity profile as of 31 December 2021 showing projections of estimated measles immunity gaps in red vertical bars for each birth cohort in a hypothetical country if a proposed SIA targeting children aged 9–59 months was to be conducted during 1–15 December 2021 and reach 95% coverage. Notes—MCV1: the first dose of measles-containing vaccines; MCV2: the second dose of measles-containing vaccines; SIA: supplementary immunization activity.

The population immunity profiles can also be generated based on projections of routine immunization coverage and assumptions on the coverage and target age groups of proposed SIAs. Figure 3 shows an example of the hypothetical country planning to conduct an SIA among children aged 9–59 months in December 2021 that should reach 95% coverage. This SIA should close the immunity gap among children less than 5 years of age. However, children born in 2014 and 2016, aged 5–7 years by the end of 2021, will still have persistent immunity gaps and will likely contribute to the risk of measles outbreaks. An expanded SIA target age group or additional vaccination activities would be needed to reduce immunity gaps among these birth cohorts.

### 3.3. Monitor the Timing of Preventive SIAs

**Figure 4:**
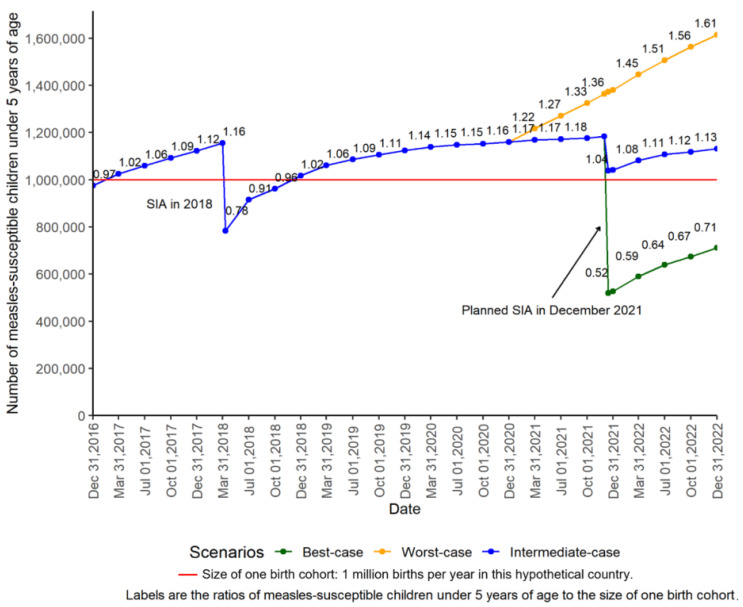
Estimates of the number of measles-susceptible children less than 5 years of age under different projected scenarios in a hypothetical country, 2016–2022. Notes—MCV1: the first dose of measles-containing vaccines; MCV2: the second dose of measles-containing vaccines; SIA: supplementary immunization activity. Best-case scenario: MCV1 and MCV2 coverage in 2021 remained the same as in 2020, and an SIA was conducted in December 2021, reaching 95% coverage. Intermediate scenario: MCV1 and MCV2 coverage in 2021 remained the same as in 2020, and an SIA was conducted in December 2021, reaching 80% coverage. Worst-case scenario: MCV1 and MCV2 coverage in 2021 decreased by 25% compared to 2020, and no SIA is conducted in 2021.

The measles immunity profile method can be used to estimate the number of measles-susceptible children less than 5 years of age at different time points and under different scenarios of coverage assumptions. Figure 4 shows the estimated number of measles-susceptible children under 5 years of age and the ratio of it to the size of one birth cohort during 2016–2022 in the hypothetical country. In this country, the number of measles-susceptible children under 5 years of age exceeded the number of one birth cohort in the first quarter of 2017. A national SIA was conducted in 2018, and the post-campaign coverage survey showed that the SIA reached 82% coverage among the target population. The ratio of the number of measles-susceptible children under five years old to the number of children in the youngest one-year birth cohort, that is, children under 1 year of age, decreased to 0.78 right after the SIA in 2018 but increased steadily because of suboptimal routine immunization coverage. The ratio exceeded 1 again in the last quarter of 2018. By the end of 2020, the ratio increased to 1.16, indicating elevated risk of measles outbreaks. If MCV1 and MCV2 coverage in 2021 remains at the same level as in 2020 and a national SIA was to be conducted in December 2021 with 95% coverage, then the number of measles-susceptible children would be reduced to about half the size of one birth cohort (best-case scenario). If the SIA reaches 80% coverage, then the number of measles-susceptible children less than 5 would remain above the size of one birth cohort (intermediate scenario). If MCV1 and MCV2 decreases by 25% in 2021 compared to 2020 (i.e., possibly due to vaccine stockout or service interruptions related to the COVID-19 pandemic) and no SIA was conducted (worst-case scenario), then the number of measles-susceptible children less than 5 years of age would increase to 1.6 million, 1.6 times the size of one birth cohort, by the end of 2022. This demonstrates the increased risk if no SIA is conducted or if an SIA is of suboptimal quality.

## 4. Discussion

We developed a measles population immunity profile tool set that builds on previous efforts by the public health community to analyze measles immunity gaps by birth cohort. The tool set promotes a standard way of analyzing measles immunity with documented methods and assumptions. Users may choose from the set of tools using different platforms, including R codes embedded in a Shiny R dashboard, or calculations in Microsoft Excel spreadsheets, based on the appropriate use case scenarios and their software skills. The profiles could be made available to partners and program managers through a web-based data visualization platform such as Power BI, helping them to identify cohorts at high risk of measles outbreaks, and to plan for preventive actions. The tool has been used to support multiple countries in the assessment of the risk of measles outbreaks, to forecast optimal timing of the next preventive SIA, and to demonstrate the needs for SIAs to prevent outbreaks. Early planning of the timely preventive SIA is critical in preventing measles outbreaks as it allows countries and donors to have sufficient lead time to allocate, approve, and secure in-country funds and to conduct micro-planning and readiness assessments to ensure quality of the SIAs. Profiles are also used to help justify to donor organizations the need for funding to support preventive SIAs.

Using our recommended assumption that MCV2 doses delivered through routine immunization and MCV doses administered during SIAs are primarily given to persons who have been previously vaccinated provides a conservative estimate of population immunity. This approach avoids overestimates that could lead to complacency, delays in vaccination activities, and preventable outbreaks and deaths. The identification of population immunity gaps allows for early planning of immunization activities to close immunity gaps. Previous research has proposed ways to quantify the efficiency of immunization activities in reaching additional children, or the percentage of zero-dose children reached based on empirical data [17,18]. The assumption on the overlap between routine doses and SIAs will be informed by additional data collected during SIAs and surveys.

The results of the measles immunity profile should be interpreted along with other information, such as measles surveillance data, to provide stronger evidence to guide programmatic decisions. The measles immunity profiles analyze susceptibility by birth year in the time dimension. It is complementary to the Measles Programmatic Risk Assessment tool, which aims to identify subnational areas at risk of measles outbreaks in the geographical dimension based on administrative coverage data only from the past three years [19]. Subnational profiles can also be constructed, provided that the required input data are available for the subnational unit(s). The tool could be used to evaluate the options on when to conduct an SIA to reduce the risk of measles outbreaks through estimating the build-up of measles-susceptible children based on projected future coverage. The tool could also be used to estimate the impact of immunization programs, such as estimating the number of additional persons immune from an SIA.

One limitation of the measles population immunity profile is that most profiles are generated for the national level and do not account for geographical variations within a country. While immunity profiles could be developed for subnational areas, such analyses are often not feasible because of the unavailability of coverage estimates validated by surveys for routine immunization or SIAs and population data at subnational levels. Because of the lack of reliable coverage and population data at subnational levels, the impact of each SIA is averaged at the national level, which is equivalent to assuming a complete mixing of the population in the country after each immunization opportunity, whether it is routine immunization or SIAs. This assumption may not hold in countries with large disparities in immunization program performance at subnational levels. Furthermore, our methods to produce an immunity profile do not consider immunity acquired through natural measles infection, likely a significant source of immunity in older age groups that were born when measles was highly endemic. The accuracy of the analysis also relies on the quality of input data. Investing in activities to obtain more accurate data, such as strengthening the availability and retention of home-based immunization records, frequent population-based health surveys including immunization indicators, and timely implementation of PCCSs, will help to improve the accuracy of the estimation of measles susceptibility.

Future development of this tool may include linking the measles population immunity profiles with measles dynamic models to account for the proportion of persons immune from natural measles infection and validating if the population immunity gaps estimated using this method are predictive of measles outbreaks detected through surveillance. More training materials and training opportunities will be provided to build capacity among immunization program managers and technical partners to use the tool for programmatic purposes. Feedback from the users will be gathered to improve the usability and performance of the tools.

## 5. Conclusions

The updated methods and tools to develop the measles immunity profiles promote a standard approach of analyses with the transparent documentation of methods and assumptions. The R version of the tool generates more granular profiles. The tools can be used to identify the cohorts with large immunity gaps and at risk of measles outbreaks and demonstrate the needs for SIAs.

## Figures and Tables

**Table 1:** Assumptions and methods of previous measles population immunity profiles and the updated the Microsoft Excel 365 and R versions of the tool.

	Previous Tools		Tools Described in this Manuscript	
	**Informal versions of Excel tools**	**Measles strategic planning tool**	**Excel version**	**R version ** (in Shiny R app and Jupyter notebook, Microsoft Power BI dashboard)
Main considerations	Easy to understand calculations in an Excel workbook that allows users to estimate the number of susceptible individuals in each birth cohort.	Estimate effectiveness and cost-effectiveness of vaccination strategies.	Calculations laid out, allowing the average Microsoft Excel user to follow them.	Provide profiles more accurately reflecting the timing of vaccination opportunities and age-specific SIA VE compared to the Excel version. Possible to rapidly generate profiles for all countries.
**Assumptions and methods**				
Correlation between doses	Independence between campaign and routine immunization doses.	MCV2 reaches 25% of children who missed MCV1 if MCV2 is given before 3 years of age in the national schedule, and 75% of children who missed MCV1 if it is given after 3 years of age. SIA and MCV2 are independent of each other.	Only the dependence assumption is implemented in this version; that is, MCV2 and SIA doses are given first to children who have been previously vaccinated, and when coverage is higher than the coverage of all previous MCV vaccination opportunities, then doses are given to previously unvaccinated children.	Dependence or independence options are available, allowing users to see and compare profiles calculated using either assumption. The total dependence is the recommended default assumption.
Temporal granularity	Routine and SIA coverage applied to whole year birth cohorts. For example, an SIA in 2020 with a target age range of 9–59 months would cover 2016–2019 birth cohorts.	Routine and SIA coverage applied to whole year birth cohorts.	Routine and SIA coverage applied to whole year birth cohorts.	Routine and SIA coverage applied to the number of children born each day that are eligible for vaccination, based on the routine immunization schedule and SIA implementation dates and target age group.
Vaccine effectiveness (VE)	Non-standardized, depending on the user input.	85% for ages < 1 year, 95% for ages ≥ 1 year.	VE is dependent on age of administration: 58% for MCV1 given at 6–8 months of age or earlier, 84% at 9–11 months, 92.5% at ≥12 months of age, and 95% for MCV2 given at ≥12 months of age. For SIAs, the same VE (95%) is used for all age groups targeted by the SIA.	VE for MCV1 and MCV2: same as the Excel version. Age-specific VE estimates used for SIAs: 58% for children 6–8 months of age, 84% for children 9–11 months of age, and 95% for children ≥12 months of age.
Sequence of routine and SIA doses	Assumes SIA doses are always given after MCV1 and MCV2.	Assumes SIA doses are always given after MCV1 and MCV2.	Assumes SIA doses are always given after MCV1 and MCV2.	Routine doses and SIA doses are put in sequence according to age of administration for each birth cohort at the time of SIA implementation. For example, SIA targeting 6–59 months of age will deliver vaccine to some children before MCV1, between MCV1 and MCV2, or after MCV2 based on age of eligibility according to the national immunization schedule.
Subnational SIAs and phased SIAs	Subnational SIAs in the same year are combined to calculate the average coverage in the proportion of the country that is covered. Phased SIAs over multiple years are combined into one entry, and the year when the greatest proportion of target population is reached is assigned as the year of implementation for the combined SIA.	Subnational SIAs in the same year are combined to calculate the average coverage in the proportion of the country that is covered. Phased SIAs over multiple years are combined into one entry, and the year when the greatest proportion of the total target population is reached is assigned as the year of implementation for the combined SIA.	Subnational SIAs in the same year are combined to calculate the average coverage in the proportion of the country that is covered. Phased SIAs over multiple years are combined into one entry, and the year when the greatest proportion of the total target populations is reached is assigned as the year of implementation for the combined SIA.	The percentage of additional children reached is calculated for each subnational area covered based on the exact dates when the subnational/phased SIAs were conducted, and then aggregated at the national level for a national profile.
Immunity acquired through natural infection	Focused on immunity among younger children. The method does not estimate immunity acquired through natural infection, although assumptions may be made on the percentage of older populations immune through natural infection.	A dynamic transmission model was used to estimate immunity acquired through natural infections. Profiles with and without immunity through natural infection are generated.	Focused on immunity among younger children. The method does not estimate immunity acquired through natural infection.	Focused on immunity among younger children. The method does not estimate immunity acquired through natural infection.

Notes—MCV1: the first dose of measles-containing vaccines; MCV2: the second dose of measles-containing vaccines; SIA: supplementary immunization activity; VE: vaccine effectiveness.

**Table 2:** Annual coverage of the first and second dose of measles-containing vaccine (MCV1 and MCV2, respectively) in a hypothetical country, 2000–2020.

Year of Vaccine Administration	MCV1	MCV2
2020	82	70
2019	81	68
2018	79	65
2017	85	60
2016	82	54
2015	80	23
2014	82	0
2013	80	0
2012	76	0
2011	75	0
2010	80	0
2009	81	0
2008	85	0
2007	84	0
2006	82	0
2005	81	0
2004	80	0
2003	75	0
2002	74	0
2001	74	0
2000	72	0

Notes—MCV1: the first dose of measles-containing vaccines; MCV2: the second dose of measles-containing vaccines.

**Table 3:** Supplementary immunization activities using measles-containing vaccines since 2000 in a hypothetical country.

Year	Intervention	Start Date	End Date	Age Group	Extent	Status	Target Population	Reached Population	% Reached	Survey Results
2007	Measles	4/1/2007	4/8/2007	6–59 M	National	Completed	4,500,000	4,140,000	92	
2010	Measles	8/12/2010	8/20/2010	9–59 M	National	Completed	4,250,000	4,037,500	95	86
2012	Measles	8/21/2012	9/4/2012	6–59 M	Subnational	Completed	2,390,000	2,103,200	88	
2014	MR	12/31/2014		9 M–14 Y	Rollover- National	Completed	10,260,000	9,336,600	91	
2015	MR	5/18/2015		9 M–14 Y	Rollover- National	Completed	3,990,000	3,710,700	93	
2016	MR	2/21/2016	3/5/2016	6–59 M	Subnational	Completed	1,090,000	1,079,100	99	
2018	MR	4/1/2018	4/10/2018	6–59 M	National	Completed	4,500,000	4,050,000	90	82
2021	MR	12/1/2021	12/15/2021	9–59 M	National	Planned	4,250,000			

Notes—M: months; Y: years. Date format: MM/DD/YYYY.

## Data Availability

Vaccination coverage and population data presented in this study were hypothetical. When developing national level profiles, we typically use WHO-UNICEF Estimates of National Immunization Coverage (available at https://immunizationdata.who.int/, accessed 16 August 2024), population data from United Nations Population Division (available at https://population.un.org/wpp/, accessed 8 August 2024), or data provided by national governments.
